# Impact of Biological Feedback and Incentives on Blood Fatty Acid Concentrations, Including Omega-3 Index, in an Employer-Based Wellness Program

**DOI:** 10.3390/nu9080842

**Published:** 2017-08-05

**Authors:** Michael I. McBurney, Julia K. Bird

**Affiliations:** 1 DSM Nutritional Products, 45 Waterview Blvd, Parsippany, NJ 07054, USA; michael.mcburney@dsm.com; 2 DSM Nutritional Products, Alexander Fleminglaan 1, 2613AX Delft, The Netherlands

**Keywords:** eicosapentaenoic acid (EPA), docosahexaenoic acid (DHA), arachidonic acid (AA), omega-3 fatty acids, omega-6 fatty acids, omega-3 index, highly unsaturated fatty acids (HUFA), EPA:AA

## Abstract

Eicosapentaenoic acid (EPA, C20:5n-3) and docosahexaenoic acid (DHA, C22:6n-3) are important fatty acids for the retina and brain. More than 95% of Americans have suboptimal EPA + DHA blood concentrations. This cross-sectional employer-based study assessed whole blood fatty acid levels of volunteers participating in an onsite wellness biometric screening program and was designed to determine if an incentive, a $5 coupon for a 90-day supply of fish oil supplement typically costing $18–30, stimulated incremental dietary behavior change relative to nutritional status assessment alone to increase EPA + DHA concentrations. Volunteers completed a dietary survey and finger stick blood samples were collected to be analyzed for fatty acid composition. In addition, 636 individuals participated in the initial onsite biometric screening. Three months later, and without prior knowledge, all employees were invited to a second screening. At the second screening, 198 employees volunteered for the first time and 149 employees had a second test (17.9%). At baseline, the average age (*n* = 834) was 45 year and omega-3 index was 5.0% with 41% female. EPA + DHA concentration, i.e., omega-3 index, was significantly lower in men (4.8%) than women (5.2%), as were DHA and linoleic acid (LA) concentrations (*p* < 0.05). Baseline omega-3 index was positively and linearly associated with omega-3 intake. Only 4% of volunteers had an omega-3 index >8% on initial screening. Among the 149 individuals with two measurements, omega-3 intake from supplements, but not food, increased significantly from 258 to 445 mg/d (*p* < 0.01) at the second test as did the omega-3 index (+0.21, *p* < 0.02). In this employed population, only 1% redeemed a coupon for an omega-3 supplement.

## 1. Introduction

Fat is a major source of energy. Evidence suggests that human health may be affected by quantity and types of fatty acids being consumed [1]. Only two fatty acids, α-linolenic acid (ALA, C18:3n-3) and linoleic acid (LA, C18:2n-6), are known to be essential for physiological and structure functions [2]. ALA and LA are also the principal n-3 and n-6 unsaturated fatty acids found in the western diet. Adequate Intakes (AI) have been set for ALA (1.6 g/d) and (1.1 g/d) and for LA (17 g/d) and (12 g/d) in men and women, respectively [3].

Within the body, ALA and LA are desaturated and elongated by shared enzymes to longer chain highly unsaturated fatty acids (HUFA), including the eicosanoids: prostaglandins, thromboxanes and leukotrienes [4]. The conversion rate of ALA to eicosapentaenoic acid (EPA, C20:5n-3) and then to docosahexaenoic acid (DHA, C22:6n-3) is very low [5], influenced by the proportions of ALA and LA in the diet [6], and lower in men than women [5]. With increased consumption of vegetable oils high in LA (corn, soybean, sunflower, safflower, and cottonseed oil), dietary imbalances in n-6 to n-3 content are hypothesized to contribute to chronic overproduction of arachidonic acid (AA, C20:4n-6) derived eicosanoids [7,8,9]. EPA:AA imbalances may promote inflammation [10,11,12] and increase cardiovascular risk [13,14,15]. 

Ingesting greater amounts of dietary EPA and DHA increases concentrations in blood [16,17]. The cytosolic phospholipase A2 (cPLA2) enzyme does not appear to discriminate between AA or EPA [18]. Therefore, increasing circulating EPA + DHA concentrations by consuming more fatty fish and/or omega-3 supplements changes the production of bioactive eicosanoids and may provide more DHA to the brain and eye [16,17,19]. 

Data from the Food4Me European randomized controlled trial suggests that personalized interventions may promote larger, more appropriate, and sustained changes in dietary behavior and health outcomes [20]. Given the suboptimal EPA + DHA status of most Americans [21], objectives of this cross-sectional employer-based study were: (1) to assess whole blood fatty acid levels of volunteers participating in an onsite wellness biometric screening program and (2) to determine if an incentive, a $5 coupon for a 90-day supply of omega-3 supplements, leads to incremental dietary behavior changes that affect blood EPA + DHA concentrations.

## 2. Materials and Methods

This study was approved by the New England Independent Review Board, a WIRB-Copernicus Group Company, Needham, MA, USA (NEIRB #20160533) and registered at clinicaltrials.gov as NCT02883764. Employees of Royal DSM company working at one of many sites throughout the United States can reduce employee-based health insurance costs by having annual biometric screenings. Approximately 40% of employees at 16 locations typically participate in a worksite biometric screening program, Healthyroads Wellness^®^, that includes a finger stick to measure blood cholesterol concentrations. The date and location of the Healthyroads Wellness^®^ biometric screening are disseminated by email and signs are posted in common areas. In 2016, the email announcement (Figure S1) included a copy of this omega-3 fatty acid research proposal and people were encouraged to have whole blood fatty acid concentrations measured. 

Upon arrival for their Healthyroads Wellness^®^ screening, employees volunteering to have blood fatty acid levels measured signed the Informed Consent (Figure S2), completed the Test Request (Figure S3) with a validated food-frequency dietary survey for assessing long-chain n-3 polyunsaturated fatty acids [22], and labeled a filter paper (Figure S4) before proceeding to the biometric screening station. 

Based on workplace demographics, number of employees, male/female distribution, and salaried/hourly proportions, the 16 worksites were categorized so that all employees volunteering at seven of the 16 worksites would receive a coupon ($5 value) for a 90 day supply of fish oil capsules (1400 mg fish oil, 900 mg omega-3 fatty acids, 647 mg EPA + 253 mg DHA per capsule) redeemable from a national retailer (Figure S5). Volunteers from the remaining nine sites were not offered a coupon.

A single drop of scavenged whole blood was collected directly to a filter paper (Ahlstrom 226, PerkinElmer, Greenville, SC, USA) pretreated with an antioxidant cocktail (FAPS™, OmegaQuant Analytics, LLC, Sioux Falls, SD, USA) to protect unsaturated fatty acids (FAs) from oxidation. After collection, the filter paper, Informed Consent, and Test Request form from each volunteer were stapled together. Materials from each worksite were shipped in bulk at ambient temperature to OmegaQuant Analytics LLC where blood samples were analyzed by capillary gas chromatography and the omega-3 index was determined [23]. OmegaQuant Analytics LLC sent an Omega-3 Index Report to the email address provided by each volunteer (Figure S6).

Three months after the Healthyroads Wellness^®^ screening, all employees received an email, including a copy of the research protocol, informing them of a second opportunity to have whole blood fatty acids measured onsite. Informed consent, test request with validated dietary survey, and blood samples collected from volunteers were sent to OmegaQuant Analytics LLC as described above. 

OmegaQuant Analytics collated and anonymized dietary survey and fatty acid data. The percent n-6 in HUFA was calculated according to the equation given in citation [9] except that C20:3n-9 was not measured. Differences between baseline and retest measurements were compared for significance using *t*-tests. Simple linear regression was used to assess the linear relationship between variables. To contrast multiple groups, a one-way ANOVA was performed. The level of significance was set at 0.05, adjusted with the Bonferroni correction for multiple analyses. Statistical analyses were conducted using SAS (Version 9.3, Durham, NC, USA). 

## 3. Results

**Figure 1:**
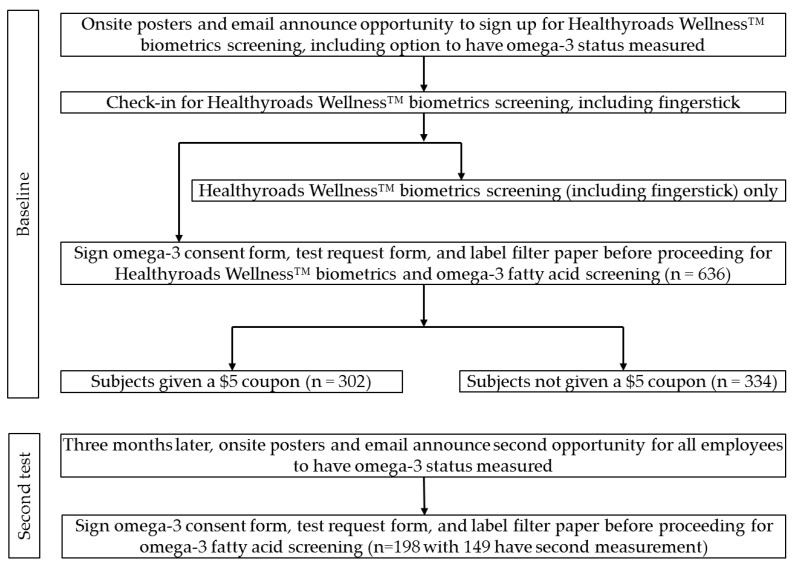
The flow diagram describing enrollment and participation in omega-3 screening.

The cross-sectional study was designed to test the impact of receiving personalized nutritional assessment with or without an incentive ($5 coupon for 90-day supply of omega-3 supplements) on omega-3 fatty acid status three months later in an employed population who was unaware a second assessment would be available (Figure 1). 

### 3.1. Cross-Sectional Results

Initially, 636 individuals participated in the first biometrics screening with 302 (47.5%) receiving coupons. The coupon manufacturer provided coupons with a bar code that are scanned upon redemption at all retail locations. The vendor tracked the number of coupons redeemed. Only three coupons were redeemed and data from all worksites have been combined. An additional 198 individuals were tested three months later and 149 participants were retested for the second time. In total, 834 individuals, with an average age of 45 years and 41% female took part in the baseline biometrical testing (Table 1). 

The average omega-3 intake from food was 254 ± 9 mg/day (Table 2). Omega-3 supplement users (24% of the study population) had higher total omega-3 intakes (965 ± 53 mg/day) than non-users (248 ± 12 mg/day). 

Only 37% of subjects were consuming ≥250 mg EPA + DHA from food sources daily. The average baseline omega-3 index was 5.0% (Table 3) and 95.8% of participants were below the recommended 8% [24]. The omega-3 index was significantly lower in men (4.8% ± 0.07) than women (5.2% ± 0.10, *p* < 0.0005), as were LA and DHA concentrations (Table 3). 

**Figure 2:**
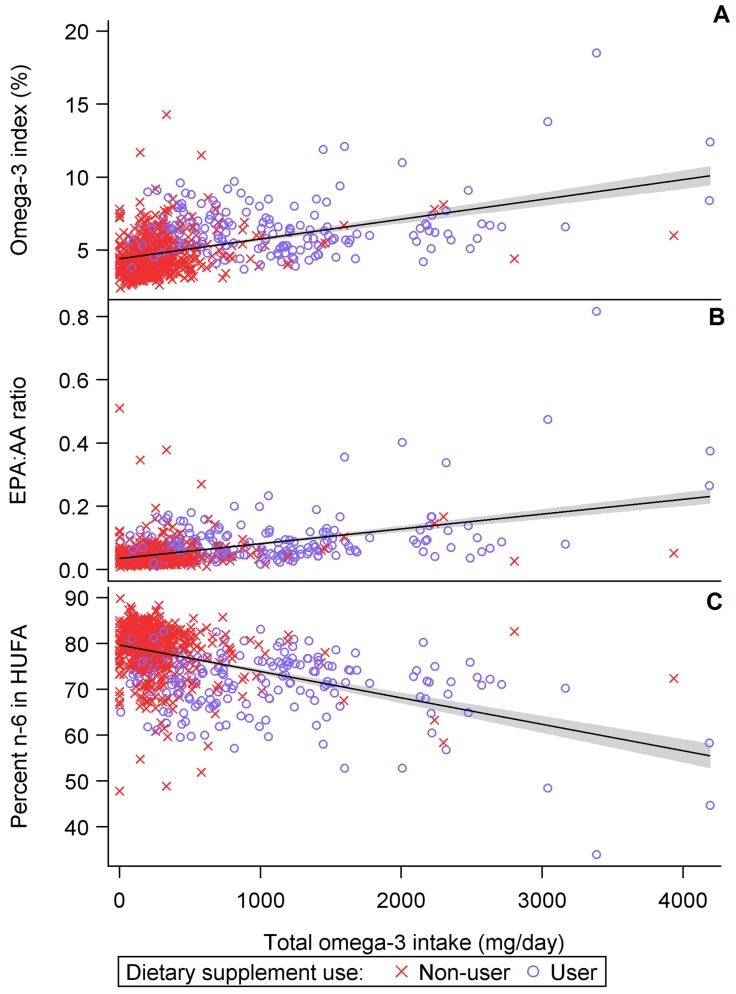
Blood fatty acid measures versus omega-3 intake by omega-3 supplement use at baseline (*n* = 834) for (**A**) omega-3 index or (**B**) EPA:AA ratio or (**C**) percent n6 in HUFA.

The omega-3 index and EPA:AA ratio showed a positive, linear relationship whereas the percent n-6 in HUFA was inversely related to omega-3 intake (Figure 2). 

### 3.2. Subgroup Analysis of Baseline and Second Test Results

Baseline and second test results were obtained for 149 individuals (17.9%). In this subset, total omega-3 intake increased from 508 ± 45 mg/d at baseline to 650 ± 65 mg/d (*p* < 0.02) at the second test, primarily because of a significant change in omega-3 supplement intake (Table 4). 

Food omega-3 intake did not change between tests. Men, but not women, reported significantly greater total omega-3 intake at the second test with this difference attributable to omega-3 supplementation. As a result, there was a significant positive change in the omega-3 index and EPA:AA ratio and a decrease in the percent n-6 in HUFA (Table 5). 

### 3.3. Subgroup Analysis Categorized by Omega-3 Supplement Use

With two assessments, the baseline and second test, individuals could be categorized according to omega-3 supplement usage at the baseline and second test: No-No (Non-users), No-Yes (Adopters), Yes-No (Discontinuers) and Yes-Yes (Users). Omega-3 index values increased with omega-3 supplement use (Adopters and Users) between the baseline and the second test (Table 6) as expected from intervention trials [17,25]. A statistically significant increase was observed among Users (*p* < 0.05). At the second test, omega-3 index values were lowest for Non-users (4.5%), then Discontinuers (5.4%), Adopters (5.6%) and Users (6.7%) (*p* < 0.05). Adopters had significantly higher omega-3 index (4.8% at baseline vs. 5.6% at the second test, *p* = 0.02). EPA:AA ratios increased among Adopters and Users (Table 6) whereas percent n-6 in HUFA decreased.

**Figure 3:**
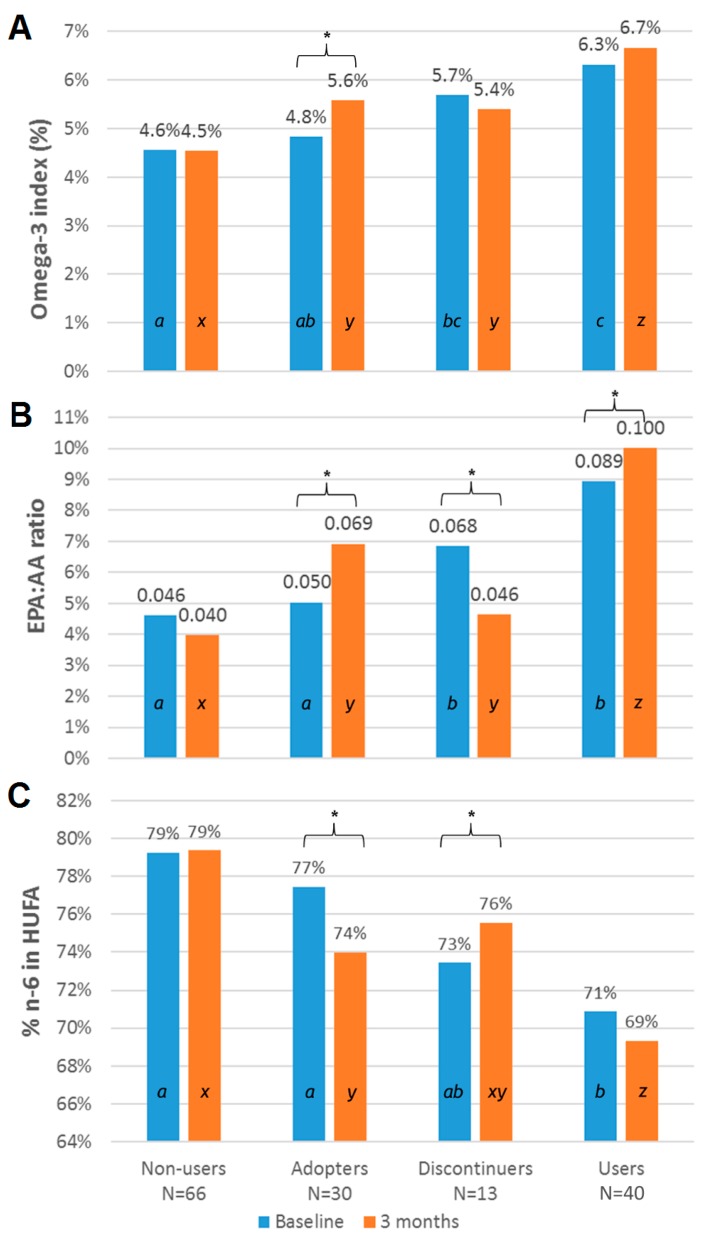
Blood fatty acid measures versus omega-3 intake according to omega-3 supplement use at baseline and three months (n = 149) for (**A**) omega-3 index or (**B**) EPA:AA ratio or (**C**) percent n6 in highly unsaturated fatty acids (HUFA). Statistical differences (*p* < 0.05) among means for Non-Users, Adopters, Discontinuers, and Users within a blood fatty acid measurement (**A**, **B**, or **C**) at baseline are denoted by a, b, c and at three months by x, y, z. * denotes a significant difference within an omega-3 use category between baseline and three months.

Average baseline omega-3 index values were significantly higher with omega-3 supplement use at baseline (Discontinuers and Users at 6.3% vs. Non-users and Adopters at 4.6% (*p* < 0.05, Figure 3A). Baseline omega-3 supplement use was associated with higher EPA:AA ratios when omega-3 supplements were being used (Figure 3B, *p* < 0.05). As expected, percent n-6 in HUFA was highest among those who do not use omega-3 supplements at baseline (Figure 3C, *p* < 0.05). 

## 4. Discussion

Low omega-3 intakes (<250 mg/day) are estimated to contribute to 55,000 coronary heart disease deaths in the United States annually [26]. We observed an average EPA + DHA intake from food of ~250 mg/d. Similar to nationally representative U.S. [21] and Canadian data [27], <5% of the study population had an omega-3 index above 8%, the level recommended for cardiovascular health [24]. Based on published data [28], an additional 850–900 mg/d of EPA + DHA is needed to raise the omega-3 index from the 5.0% group average to 8.0%. 

Omega-3 index values were significantly higher in women than men, 5.2 ± 0.1 vs. 4.8 ± 0.1, respectively (*p* < 0.005), as has been reported by some [17,25] but not others [29,30]. Women of reproductive age convert ALA to EPA and then DHA at higher rates than men [5]. Most (80.2% ± 1.9%) participants had percent n-6 in HUFA values below the 61% recommendation of Bibus and Lands [8]. An EPA:AA ratio above 0.3 is associated with reduced risk of cardiovascular disease [13] yet 99% ± 1% were below this target. 

In the 149 subjects with two blood tests, baseline measurement of omega-3 index had a positive dietary behavior impact on 70 individuals (Adopters and Users representing 47% of the 149 tested twice). Sixty-six Non-users (44%) with a baseline omega-3 index of 4.6% continued to avoid supplements. It would be interesting to know if providing additional educational materials on health benefits of higher omega-3 index values might stimulate this cohort to change their dietary behavior. In the 53 Users (27%) using supplements at baseline and the second test, the mean omega-3 index increased from 6.3% to 6.7%, suggesting they increased their commitment to consuming sources of long chain omega-3 fatty acids. Adopters (30%) changed dietary behavior by starting to use omega-3 supplements and had the largest increase in omega-3 index with a 17% increase (4.8% to 5.6%) between tests. Increased omega-3 supplement use is expected to raise EPA + DHA concentrations [17,25]. Higher concentrations of DHA have been reported to slow the release of both AA and EPA [31] by cPLA2, possibly explaining the smaller reduction in red blood cell AA concentrations of AA observed in this study among Users and reported with high-dose DHA vs. high-dose EPA supplementation [32]. 

This study is limited because it is a cross-sectional workplace study. A primary goal, measuring the impact of a financial incentive, failed because coupon redemption was almost non-existent. It was surprising to us that many participants refused to take a coupon at baseline testing. The financial value, choice of retailer, and consumer-brand preference could have been factors. It is also possible that coupons may have been lost or discarded in the 7–10 day period between testing and receipt of omega-3 index results by email.

## 5. Conclusions

This study corroborates national surveys [21,27] of the poor omega-3 status of Americans. In this employed group of volunteers, being handed a $5 coupon 7–10 day prior to receiving nutritional assessments was not a motivator. Only 1% of coupons were redeemed. Based upon repeat measures in 149 participants, we observed that personalized nutrition assessment, i.e., knowing your omega-3 index, motivated ~50% of people (Users and Adopters) to make dietary changes and increase their average omega-3 index. Unfortunately, as reported elsewhere [33], those with the lowest nutritional status, i.e., Non-Users representing 44% of the participants, did not change their dietary behaviors after baseline nutritional assessment and their blood fatty acid concentrations corroborate this. More research is needed to understand if different financial incentives and/or additional education and guidance at baseline testing or personalized guidance included with the omega-3 index report might have a greater impact on subsequent dietary behavior, nutritional status and health outcome. 

## Figures and Tables

**Table 1:** Characteristics of participants at baseline.

Parameter	All Participants	Participants Undergoing Retest	Participants Not Undergoing Retest
*N*	834	149	685
Sex (male), *N* (%)	490 (59%)	81 (54%)	409 (59%)
Age (year), mean (SE)	45 (0.4)	45 (0.4)	45 (0.9)
Omega-3 index, mean (SE)	5.0% (0.1%)	5.2% (0.1%)	4.9% (0.1%)
Percent n-6 in HUFA, mean (SE)	77.2% (0.2%)	76.1% (0.3%)	77.5% (0.5%)
EPA to AA ratio	0.054 (0.001)	0.060 (0.004)	0.053 (0.002)
Baseline omega-3 index category, *N* (%)			
<4%	223 (27%)	32 (21%)	191 (28%)
4–8%	576 (69%)	109 (73%)	467 (68%)
>8%	35 (4.2%)	8 (5.4%)	27 (3.9%)
Dietary supplement use, *N* (%)	205 (25%)	53 (36%)	152 (22%)

**Table 2:** Mean omega-3 intake (mg/day) at baseline.

	All Participants	Dietary Supplement Users	Non-Users	Male	Female
*N*	834	205	628	490	344
Food	254 ± 9.4	271 ± 14	248 ± 12	256 ± 13	250 ± 14
Supplement	180 ± 17	867 ± 56	0 ± 0	187 ± 21	170 ± 29
Total	424 ± 19	965 * ± 53	248 ± 12	434 ± 23	410 ± 31

* For some Dietary Supplement Users, the dose taken was unreadable, therefore the total omega-3 intake does not equal food and supplement sum.

**Table 3:** Participant whole blood fatty acid values at the baseline.

Fatty Acid	All Participants	Men	Women	*p*-Value *
*N*	834	490	344	-
Omega-3 index	5.0 ± 0.06	4.8 ± 0.07	5.2 ± 0.10	**0.0005**
EPA:AA ratio	0.054 ± 0.00	0.052 ± 0.00	0.057 ± 0.00	0.13
n-6 in HUFA (%)	77 ± 0.2	78 ± 0.3	77 ± 0.4	0.03
C14:0 (%)	0.63 ± 0.01	0.66 ± 0.01	0.60 ± 0.01	0.0025
C16:0 (%)	21.9 ± 0.1	22.0 ± 0.07	21.8 ± 0.1	0.09
C16:1n7t (%)	0.12 ± 0.00	0.12 ± 0.00	0.12 ± 0.00	0.8
C16:1n7 (%)	0.94 ± 0.02	0.90 ± 0.02	0.98 ± 0.03	0.029
C18:0 (%)	11.7 ± 0.04	11.7 ± 0.05	11.5 ± 0.06	0.027
C18:1t (%)	0.61 ± 0.01	0.62 ± 0.01	0.58 ± 0.01	0.0091
C18:1n9 (%)	18.6 ± 0.1	19.0 ± 0.1	18.1 ± 0.1	**0.0001**
C18:2n6t (%)	0.24 ± 0.00	0.23 ± 0.01	0.25 ± 0.01	0.0305
C18:2n6 (%)	22.9 ± 0.1	22.6 ± 0.1	23.4 ± 0.1	**0.0001**
C20:0 (%)	0.22 ± 0.00	0.21 ± 0.00	0.23 ± 0.00	**0.0001**
C18:3n6 (%)	0.29 ± 0.00	0.30 ± 0.01	0.28 ± 0.01	0.019
C20:1n9 (%)	0.28 ± 0.00	0.28 ± 0.01	0.29 ± 0.01	0.14
C18:3n3 (%)	0.40 ± 0.01	0.41 ± 0.01	0.39 ± 0.01	0.23
C20:2n6 (%)	0.24 ± 0.00	0.23 ± 0.00	0.26 ± 0.00	**0.0001**
C22:0 (%)	0.49 ± 0.01	0.47 ± 0.01	0.51 ± 0.01	**0.0001**
C20:3n6 (%)	1.6 ± 0.01	1.5 ± 0.01	1.6 ± 0.02	0.068
C20:4n6 (%)	11.0 ± 0.1	11.0 ± 0.08	11.0 ± 0.1	0.71
C24:0 (%)	0.7 ± 0.0	0.7 ± 0.0	0.7 ± 0.0	0.61
C20:5n3 (%)	0.58 ± 0.02	0.55 ± 0.02	0.61 ± 0.03	0.11
C24:1n9 (%)	0.61 ± 0.01	0.58 ± 0.01	0.65 ± 0.02	**0.0004**
C22:4n6 (%)	1.6 ± 0.01	1.6 ± 0.02	1.5 ± 0.02	0.004
C22:5n6 (%)	0.36 ± 0.01	0.36 ± 0.01	0.37 ± 0.01	0.27
C22:5n3 (%)	1.2 ± 0.01	1.2 ± 0.01	1.1 ± 0.01	**0.0001**
C22:6n3 (%)	2.6 ± 0.04	2.5 ± 0.04	2.8 ± 0.06	**0.0001**

* *p*-value for the difference between sexes, bold if less than 0.0017 (*p* = 0.05 with Bonferroni correction for 28 tests).

**Table 4:** Omega-3 intake (mg/day) at the baseline and second test for individuals undergoing two screenings three months apart.

	*N*	Food	Dietary Supplements	Total
All				
Baseline	149	263 ± 17	258 ± 42	508 ± 45
Retest	149	253 ± 15	445 ± 69	650 ± 65
*p*-value ^1^		>0.05	0.0136	0.0185
Men				
Baseline	81	276 ± 195	385 ± 609	638 ± 611
Retest	81	273 ± 199	640 ± 965	850 ± 946
*p*-value ^2^		>0.05	0.039	0.039
Women				
Baseline	68	248 ± 222	110 ± 275	354 ± 408
Retest	68	228 ± 164	208 ± 434	412 ± 481
*p*-value ^2^		>0.05	>0.05	>0.05

^1^ Dietary intake, baseline vs. retest, for all participants; ^2^ Dietary intake, baseline vs. retest, within a sex.

**Table 5:** Change in fatty acid status between baseline and second test for individuals undergoing two screenings 3 months apart.

	All	*p*-Value ^1^	Men	Women	*p*-Value ^2^
N	149	-	81	68	-
Omega-3 index	0.21 ± 0.11	0.02	0.26 ± 0.13	0.15 ± 0.12	>0.05
EPA:AA ratio	0.002 ± 0.003	>0.05	0.007 ± 0.005	−0.004 ± 0.005	>0.05
Percent n-6 in HUFA	−0.884 ± 0.301	<0.004	−0.012 ± 0.004	0.009 ± 0.003	>0.05

^1^ Change calculated as baseline value minus retest value; ^2^ Change calculated as baseline value minus retest value for men and for women.

**Table 6:** Fatty acid values at baseline and second test according to dietary supplementation practices in 149 volunteers who were retested.

	Baseline					Second Test				
Fatty Acid	Non-Users	Adopters	Discontinuers	Users	*p*-Value ^1^	Non-Users	Adopters	Discontinuers	Users	*p*-Value ^2^
*N*	66	30	13	40	-	66	30	13	40	
Omega-3 index	4.6 ± 0.2	4.8 ± 0.2	5.7 ± −0.4	6.3 ± 0.2	**0.0005**	4.5 ± −0.1	5.6 ± 0.2	5.4 ± 0.4	6.7 ± 0.2	**<0.0001**
EPA:AA ratio	0.05 ± 0.01	0.05 ± 0.00	0.07 ± 0.01	0.09 ± 0.01	0.79	0.04 ± 0.00	0.07 ± 0.01	0.05 ± 0.01	0.10 ± 0.01	**<0.0001**
n-6 in HUFA (%)	79 ± 0.7	79 ± 0.8	73 ± 1.6	71 ± 0.9	0.03	79 ± 0.5	74 ± 0.9	76 ± 1.7	69 ± 1.0	**<0.0001**
C14:0 (%)	0.64 ± 0.03	0.70 ± 0.06	0.60 ± 0.08	0.62 ± 0.04	0.0025	0.60 ± 0.04	0.66 ± 0.09	0.52 ± 0.06	0.65 ± 0.05	0.54
C16:0 (%)	22.0 ± 0.2	22.1 ± 0.3	22.1 ± 0.5	21.7 ± 0.2	0.09	22.0 ± 0.2	22.0 ± 0.4	21.6 ± 0.4	21.8 ± 0.3	0.84
C16:1n7t (%)	0.13 ± 0.01	0.13 ± 0.01	0.09 ± 0.01	0.12 ± 0.01	0.8	0.12 ± 0.00	0.11 ± 0.01	0.10 ± 0.00	0.12 ± 0.01	0.28
C16:1n7 (%)	0.98 ± 0.1	1.10 ± 0.1	0.89 ± 0.1	0.92 ± −0.1	0.029	0.93 ± 0.1	0.92 ± 0.1	0.81 ± 0.1	0.87 ± 0.1	0.90
C18:0 (%)	11.5 ± 0.1	11.4 ± 0.2	11.4 ± 0.4	11.5 ± 0.1	0.027	12.2 ± 0.2	11.9 ± 0.3	12.2 ± 0.4	12.2 ± 0.2	0.82
C18:1t (%)	0.64 ± 0.02	0.53 ± 0.03	0.49 ± 0.06	0.56 ± 0.02	0.0091	0.59 ± 0.03	0.59 ± 0.04	0.49 ± 0.06	0.55 ± 0.03	0.29
C18:1n9 (%)	18.4 ± 0.3	19.1 ± 0.3	18.7 ± 0.6	18.4 ± 0.4	**<0.0001**	18.5 ± 0.3	19.3 ± 0.5	19.5 ± 0.6	18.9 ± 0.4	0.42
C18:2n6t (%)	0.26 ± 0.02	0.26 ± 0.03	0.22 ± 0.02	0.21 ± 0.01	0.0305	0.21 ± 0.01	0.19 ± 0.01	0.21 ± 0.02	0.18 ± 0.01	0.22
C18:2n6 (%)	23.3 ± 0.3	22.9 ± 0.5	24.1 ± 0.6	23.1 ± 0.4	**<0.0001**	22.3 ± 0.3	22.4 ± 0.5	22.2 ± 1.1	22.4 ± 0.5	0.99
C20:0 (%)	0.22 ± 0.01	0.24 ± 0.01	0.22 ± 0.02	0.23 ± 0.01	**<0.0001**	0.21 ± 0.01	0.20 ± 0.01	0.21 ± 0.02	0.19 ± 0.01	0.50
C18:3n6 (%)	0.32 ± 0.02	0.34 ± 0.02	0.27 ± 0.03	0.29 ± 0.01	0.0191	0.31 ± 0.02	0.26 ± 0.02	0.27 ± 0.04	0.25 ± 0.02	0.13
C20:1n9 (%)	0.34 ± 0.02	0.33 ± 0.03	0.25 ± 0.02	0.29 ± 0.02	0.14	0.25 ± 0.01	0.24 ± 0.01	0.28 ± 0.02	0.23 ± 0.01	0.10
C18:3n3 (%)	0.41 ± 0.02	0.39 ± 0.03	0.39 ± 0.03	0.39 ± 0.02	0.23	0.37 ± 0.02	0.41 ± 0.03	0.36 ± 0.05	0.41 ± 0.04	0.62
C20:2n6 (%)	0.24 ± 0.01	0.26 ± 0.01	0.26 ± 0.02	0.23 ± 0.01	<0.0001	0.25 ± 0.01	0.22 ± 0.01	0.24 ± 0.01	0.22 ± 0.01	0.037
C22:0 (%)	0.49 ± 0.02	0.50 ± 0.03	0.49 ± 0.04	0.53 ± 0.02	<0.0001	0.49 ± 0.02	0.44 ± 0.03	0.48 ± 0.04	0.42 ± 0.02	0.19
C20:3n6 (%)	1.6 ± 0.05	1.6 ± 0.06	1.5 ± 0.08	1.5 ± 0.06	0.068	1.6 ± 0.04	1.5 ± 0.06	1.5 ± 0.07	1.5 ± 0.06	0.15
C20:4n6 (%)	11.4 ± 0.2	10.8 ± 0.4	10.5 ± 0.5	10.8 ± 0.3	0.71	11.7 ± 0.2	10.9 ± 0.4	11.4 ± 0.6	10.7 ± 0.3	0.06
C24:0 (%)	0.63 ± 0.03	0.64 ± 0.05	0.66 ± 0.08	0.76 ± 0.05	0.61	0.68 ± 0.05	0.53 ± 0.05	0.59 ± 0.07	0.52 ± 0.04	0.06
C20:5n3 (%)	0.519 ± 0.1	0.53 ± 0.0	0.72 ± 0.1	0.91 ± 0.1	0.11	0.46 ± 0.0	0.73 ± 0.1	0.52 ± 0.1	1.04 ± 0.1	**<0.0001**
C24:1n9 (%)	0.58 ± 0.03	0.61 ± 0.06	0.61 ± 0.09	0.69 ± 0.04	**0.0004**	0.59 ± 0.04	0.47 ± 0.04	0.55 ± 0.04	0.46 ± 0.03	0.0377
C22:4n6 (%)	1.63 ± 0.05	1.49 ± 0.06	1.21 ± 0.08	1.26 ± 0.06	0.0038	1.75 ± 0.05	1.48 ± 0.08	1.45 ± 0.09	1.27 ± 0.07	**<0.0001**
C22:5n6 (%)	0.37 ± 0.01	0.35 ± 0.02	0.30 ± 0.02	0.35 ± 0.03	0.27	0.37 ± 0.01	0.31 ± 0.02	0.30 ± 0.02	0.29 ± 0.03	**0.008**
C22:5n3 (%)	1.13 ± 0.03	1.11 ± 0.04	1.13 ± 0.09	1.29 ± 0.04	**<0.0001**	1.20 ± 0.04	1.26 ± 0.06	1.22 ± 0.10	1.36 ± 0.06	0.09
C22:6n3 (%)	2.32 ± 0.1	2.54 ± 0.2	3.12 ± 0.3	3.48 ± 0.1	**<0.0001**	2.37 ± 0.1	3.01 ± 0.2	3.07 ± 0.3	3.65 ± 0.2	**<0.0001**

^1^ Difference between groups at baseline, bold if less than 0.0017 (*p* = 0.05 with Bonferroni correction for 28 tests); ^2^ Difference among groups at second test, bold if less than 0.0017 (*p* = 0.05 with Bonferroni correction for 28 tests).

## Supplementary Materials

The following are available online at www.mdpi.com/2072-6643/9/8/842/s1, Figure S1: Advertisement, Figure S2: Consent form, Figure S3: Test request form, Figure S4: Filter paper for blood spot, Figure S5: Coupon incentive, Figure S6: Sample report.

## References

[1] Raatz S.K., Conrad Z., Johnson L.K., Picklo M.J., Jahns L. (2017). Relationship of the Reported Intakes of Fat and Fatty Acids to Body Weight in US Adults. Nutrients.

[2] U.S. Institute of Medicine (2005). Dietary Reference Intakes for Energy, Carbohydrate, Fiber, Fat, Fatty Acids, Cholesterol, Protein, and Amino Acids.

[3] Kris-Etherton P.M., Grieger J.A., Etherton T.D. (2009). Dietary reference intakes for DHA and EPA. Prostaglandins Leukot. Essent. Fatty Acids.

[4] Pace-Asciak C.R., Smith W.L., Boyer P.D. (1983). 16 Enzymes in the Biosynthesis and Catabolism of the Eicosanoids: Prostaglandins, Thromboxanes, Leukotrienes and Hydroxy Fatty Acids. The Enzymes.

[5] Burdge G.C., Calder P.C. (2005). Conversion of alpha-linolenic acid to longer-chain polyunsaturated fatty acids in human adults. Reprod. Nutr. Dev..

[6] Goyens P.L., Spilker M.E., Zock P.L., Katan M.B., Mensink R.P. (2006). Conversion of α-linolenic acid in humans is influenced by the absolute amounts of α-linolenic acid and linoleic acid in the diet and not by their ratio. Am. J. Clin. Nutr..

[7] Lands B., Bibus D., Stark K.D. (2017). Dynamic interactions of n-3 and n-6 fatty acid nutrients. Prostaglandins Leukot. Essent. Fatty Acids.

[8] Bibus D., Lands B. (2015). Balancing proportions of competing omega-3 and omega-6 highly unsaturated fatty acids (HUFA) in tissue lipids. Prostaglandins Leukot. Essent. Fatty Acids.

[9] Lands B., Lamoreaux E. (2012). Using 3–6 differences in essential fatty acids rather than 3/6 ratios gives useful food balance scores. Nutr. Metab..

[10] Choque B., Catheline D., Rioux V., Legrand P. (2014). Linoleic acid: Between doubts and certainties. Biochimie.

[11] Simopoulos A.P., DiNicolantonio J.J. (2016). The importance of a balanced ω-6 to ω-3 ratio in the prevention and management of obesity. Open Heart.

[12] Blasbalg T.L., Hibbeln J.R., Ramsden C.E., Majchrzak S.F., Rawlings R.R. (2011). Changes in consumption of omega-3 and omega-6 fatty acids in the United States during the 20th century. Am. J. Clin. Nutr..

[13] Nagahara Y., Motoyama S., Sarai M., Ito H., Kawai H., Takakuwa Y., Miyagi M., Shibata D., Takahashi H., Naruse H. (2016). Eicosapentaenoic acid to arachidonic acid (EPA/AA) ratio as an associated factor of high risk plaque on coronary computed tomography in patients without coronary artery disease. Atherosclerosis.

[14] Hishikari K., Kimura S., Yamakami Y., Kojima K., Sagawa Y., Otani H., Sugiyama T., Kuwahara T., Hikita H., Takahashi A. (2015). The prognostic value of the serum eicosapentaenoic acid to arachidonic acid ratio in relation to clinical outcomes after endovascular therapy in patients with peripheral artery disease caused by femoropopliteal artery lesions. Atherosclerosis.

[15] Ninomiya T., Nagata M., Hata J., Hirakawa Y., Ozawa M., Yoshida D., Ohara T., Kishimoto H., Mukai N., Fukuhara M. (2013). Association between ratio of serum eicosapentaenoic acid to arachidonic acid and risk of cardiovascular disease: The Hisayama Study. Atherosclerosis.

[16] Allaire J., Couture P., Leclerc M., Charest A., Marin J., Lépine M.-C., Talbot D., Tchernof A., Lamarche B. (2016). Randomized, crossover, head-to-head comparison of EPA and DHA supplementation to reduce inflammation markers in men and women: The Comparing EPA to DHA Study. Am. J. Clin. Nutr..

[17] Flock M.R., Skulas-Ray A.C., Harris W.S., Etherton T.D., Fleming J.A., Kris-Etherton P.M. (2013). Determinants of Erythrocyte Omega-3 Fatty Acid Content in Response to Fish Oil Supplementation: A Dose-Response Randomized Controlled Trial. J. Am. Heart Assoc..

[18] Leslie C.C. (2004). Regulation of the specific release of arachidonic acid by cytosolic phospholipase A2. Prostaglandins Leukot. Essent. Fatty Acids.

[19] Richard C., Calder P.C. (2016). Docosahexaenoic Acid. Adv. Nutr. Int. Rev. J..

[20] Celis-Morales C., Livingstone K.M., Marsaux C.F.M., Macready A.L., Fallaize R., O’Donovan C.B., Woolhead C., Forster H., Walsh M.C., Navas-Carretero S. (2016). Effect of personalized nutrition on health-related behaviour change: Evidence from the Food4Me European randomized controlled trial. Int. J. Epidemiol..

[21] Murphy R., Yu E., Ciappio E., Mehta S., McBurney M. (2015). Suboptimal Plasma Long Chain n-3 Concentrations are Common among Adults in the United States, NHANES 2003–2004. Nutrients.

[22] Kuratko C. (2013). Food-frequency questionnaire for assessing long-chain ω-3 fatty-acid intake. Nutrition.

[23] Harris W.S., Polreis J. (2016). Measurement of the Omega-3 Index in Dried Blood Spots. Ann. Clin. Lab. Res..

[24] Harris W.S., von Schacky C. (2004). The Omega-3 Index: A new risk factor for death from coronary heart disease?. Prev. Med..

[25] Patterson A.C., Chalil A., Aristizabal Henao J.J., Streit I.T., Stark K.D. (2015). Omega-3 polyunsaturated fatty acid blood biomarkers increase linearly in men and women after tightly controlled intakes of 0.25, 0.5, and 1 g/d of EPA + DHA. Nutr. Res..

[26] Micha R., Peñalvo J.L., Cudhea F., Imamura F., Rehm C.D., Mozaffarian D. (2017). Association Between Dietary Factors and Mortality From Heart Disease, Stroke, and Type 2 Diabetes in the United States. JAMA.

[27] Langlois K., Ratnayake W.M. (2015). Omega-3 Index of Canadian adults. Health Rep..

[28] Flock M.R., Harris W.S., Kris-Etherton P.M. (2013). Long-chain omega-3 fatty acids: Time to establish a dietary reference intake. Nutr. Rev..

[29] Plourde M., Chouinard-Watkins R., Rioux-Perreault C., Fortier M., Dang M.T.M., Allard M.-J., Tremblay-Mercier J., Zhang Y., Lawrence P., Vohl M.-C. (2014). Kinetics of 13C-DHA before and during fish-oil supplementation in healthy older individuals. Am. J. Clin. Nutr..

[30] West A.L., Burdge G.C., Calder P.C. (2016). Lipid structure does not modify incorporation of EPA and DHA into blood lipids in healthy adults: A randomised-controlled trial. Br. J. Nutr..

[31] Shikano M., Masuzawa Y., Yazawa K., Takayama K., Kudo I., Inoue K. (1994). Complete discrimination of docosahexaenoate from arachidonate by 85 kDa cytosolic phospholipase A2 during the hydrolysis of diacyl- and alkenylacylglycerophosphoethanolamine. Biochim. Biophys. Acta BBA Lipids Lipid Metab..

[32] Allaire J., Harris W.S., Vors C., Charest A., Marin J., Jackson K.H., Tchernof A., Couture P., Lamarche B. (2017). Supplementation with high-dose docosahexaenoic acid increases the Omega-3 Index more than high-dose eicosapentaenoic acid. Prostaglandins Leukot. Essent. Fat. Acids PLEFA.

[33] Dickinson A., MacKay D. (2014). Health habits and other characteristics of dietary supplement users: A review. Nutr. J..

