# Supplementary material for: Impact of Biological Feedback and Incentives on Blood Fatty Acid Concentrations, Including Omega-3 Index, in an Employer-Based Wellness Program
Source: Nutrients. 2017 Aug 5;9(8):842. doi: 10.3390/nu9080842 (PMC5579635; doi:10.3390/nu9080842)
Supplement: Supplementary file 1 [file nutrients-09-00842-s001.zip › Figure S5 Coupon incentive.pdf]

Heart Health*

Available at Walgreens Stores Only

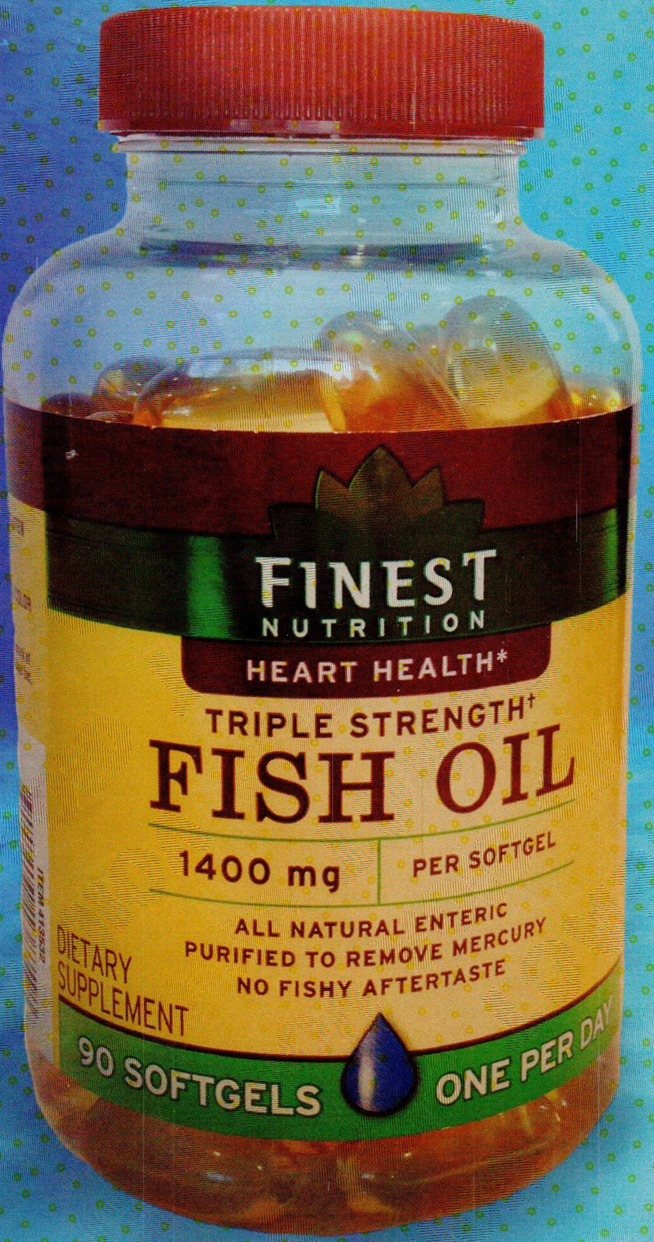

Omega-3 fatty acids are essential for the function of the heart, brain, and eyes.*

Made with EnteriCare® Technology to reduce fishy odor and aftertaste.

- Made with EnteriCare® Technology
- Made with MEG-3™ purified Fish Oil
- Gluten free
- No lactose or artificial color

MEG-3

*This statement has not been evaluated by the Food and Drug Administration. This product is not intended to diagnose, treat, cure, or prevent any disease.

Manufacturer Coupon | Expires: 09/30/16

$5 OFF

Finest Nutrition Fish Oil

0311917-005438

CONSUMER: Redeem only by purchasing the brand and size(s) indicated. May not be reproduced. Void if transferred to any person, firm, or group prior to store redemption. Any other use constitutes fraud. Consumer pays sales tax.

RETAILER: DSM Nutritional products, LLC will reimburse you the face value of thiscoupon plus 8 cents handling in accordance with our redemption policy(copy available upon request). Consumer must pay any sales tax. Send all redeemed coupons to: DSM Nutritional products, LLC Mandlik & Rhodes, PO Box 490, Dept #1502, Tecate, CA 91980. Cash value: 1/100¢.

© 2016 DSM Nutritional products, LLC, All Rights Reserved.
