# Supplementary material for: Impact of Biological Feedback and Incentives on Blood Fatty Acid Concentrations, Including Omega-3 Index, in an Employer-Based Wellness Program
Source: Nutrients. 2017 Aug 5;9(8):842. doi: 10.3390/nu9080842 (PMC5579635; doi:10.3390/nu9080842)
Supplement: Supplementary file 1 [file nutrients-09-00842-s001.zip › Figure S6 Test report.pdf]

5009 W 12th St Suite 8
Sioux Falls, SD 57106
1-800-949-0632
info@omegaquant.com
omegaquant.com

OMEGA-3 INDEX REPORT

NAME: Surname, First
DOB: mm/dd/yyyy
ID:

COLLECTION DATE: mm/dd/yyyy
RESULT DATE: mm/dd/yyyy
PROVIDER:
ACCOUNT: DSM Screening

Your Index **4.22%**

Reference Range*: 2.90% - 12.90%

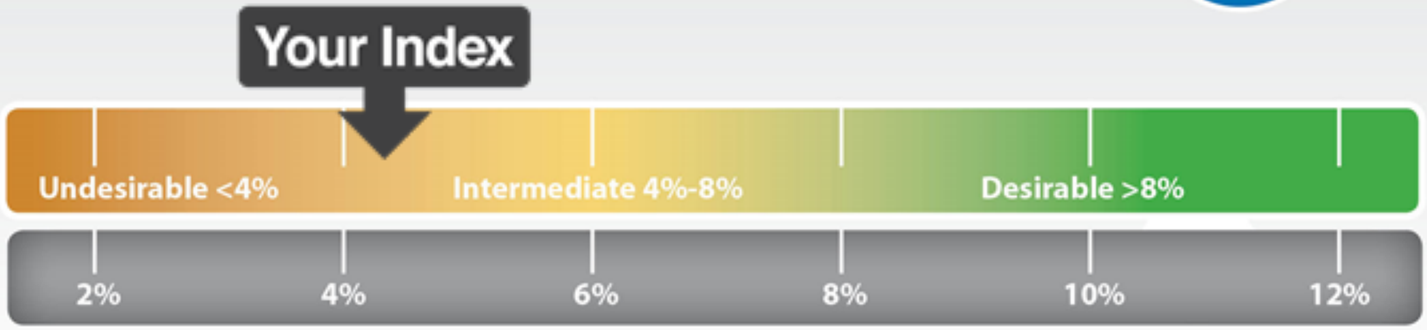

* Reference Ranges encompass about 99% of US adults. Visit our FAQ section for more information on Ratios and Ranges.

Your Omega-3 Index is in the intermediate range of 4 - 8%. You are advised to increase your intake of omega-3 fatty acids.

Many studies have shown that people with higher (vs. lower) omega-3 index levels are at decreased risk for a variety of diseases. These include heart disease, stroke, dementia, and depression to name a few. These people even live longer than those with lower levels. Raising your omega-3 index and keeping it up should help reduce your risk these conditions.

Omega-3 fatty acids are found primarily in fish, especially "oily" fish such as those near the top in the accompanying table. The two most important omega-3 fatty acids are EPA and DHA.

The amount of EPA+DHA you would need to take in order to raise your Omega-3 Index into the target range (>8%) cannot be predicted with certainty. Many factors – age, sex, weight, dietary and genetic factors, smoking, medications you may be taking, other medical conditions, etc. – all can influence your body's response to additional EPA+DHA. Nevertheless, we would recommend that you increase your current EPA+DHA intake by 0.5–1 grams (500 – 1000 mg) per day. Although this can be accomplished by eating more oily fish, fish oil supplements are usually necessary to achieve this level of EPA+DHA intake. The table lists the approximate amount of EPA and DHA per 3-oz. serving of a variety of sea foods and in dietary supplements.

It should be noted that omega-3 fatty acids from flaxseed oil (alpha-linolenic acid, or ALA) will have little to no effect on your Omega-3 Index. Therefore, ALA is not an effective substitute for EPA and DHA.

The only way to know how your body will respond to an increased intake of EPA+DHA is to measure your Omega-3 Index again. You should wait for 3-4 months before re-testing in order to give your system time to adjust to your increased intake. Once you have achieved your target Omega-3 Index you should re-check your values every six months.

Content of EPA+DHA (in mg) in Commonly Consumed Types of Fish and in Fish Oil Supplements (per 3 oz or 85 g serving size)

Fish and Seafood	EPA	DHA	EPA+DHA
Atlantic Salmon (farmed)	587	1238	1825
Pacific Herring	1056	751	1807
Atlantic Herring	773	939	1712
Atlantic Salmon (wild)	349	1215	1564
Bluefin Tuna	309	970	1279
Coho Salmon (wild)	462	706	1168
Pink Salmon (wild)	456	638	1094
Coho Salmon (farmed)	347	740	1087
Mackerel (canned)	369	677	1046
Sockeye Salmon (wild)	353	690	1043
Chum Salmon (canned)	402	597	999
Sardines (canned)	402	433	835
Pink Salmon (canned)	233	579	812
Swordfish	108	656	764
Rainbow Trout (farmed)	220	524	744
Albacore (or White) Tuna (canned)	198	535	733
Shark (raw)	269	448	717
Sea Bass	175	473	648
Atlantic Pollock	77	383	460
King Crab	251	100	351
Walleye/Pike	94	245	339
Dungeness Crab	239	96	335
Oysters (farmed, raw)	160	173	333
Skipjack Tuna	77	201	278
Flat Fish (Flounder/Sole)	143	112	255
Clams	117	124	241
Mixed Shrimp	115	120	235
Light Chunk Tuna	40	190	230
Catfish (wild)	85	116	201
Halibut	68	132	200
King Mackeral	5	193	198
Scallops	61	88	149
Blue Crab	86	57	143
Cod	3	131	134
Mahi-Mahi (Dolphin Fish)	22	96	118
Tilapia	4	110	114
Yellowfin Tuna	13	89	102
Catfish (farmed)	17	59	76
Dietary Supplements – Amount (mg) per 1,000 mg capsule or per teaspoon			
Standard Drug Store Fish Oil Capsules	180	120	300
Fish Oil Concentrates (many varieties)	100-400	100-400	300-700
Cod Liver Oil (teaspoon)	300	500	800

Table adapted from Harris et al. Current Atherosclerosis Reports 2008;10:503-509. Values based on USDA Nutrient Data Lab values and are for fish cooked with dry heat unless otherwise noted.
