# Supplementary material for: Impact of Biological Feedback and Incentives on Blood Fatty Acid Concentrations, Including Omega-3 Index, in an Employer-Based Wellness Program
Source: Nutrients. 2017 Aug 5;9(8):842. doi: 10.3390/nu9080842 (PMC5579635; doi:10.3390/nu9080842)
Supplement: Supplementary file 1 [file nutrients-09-00842-s001.zip › Figure S1 Advertisement.pdf]

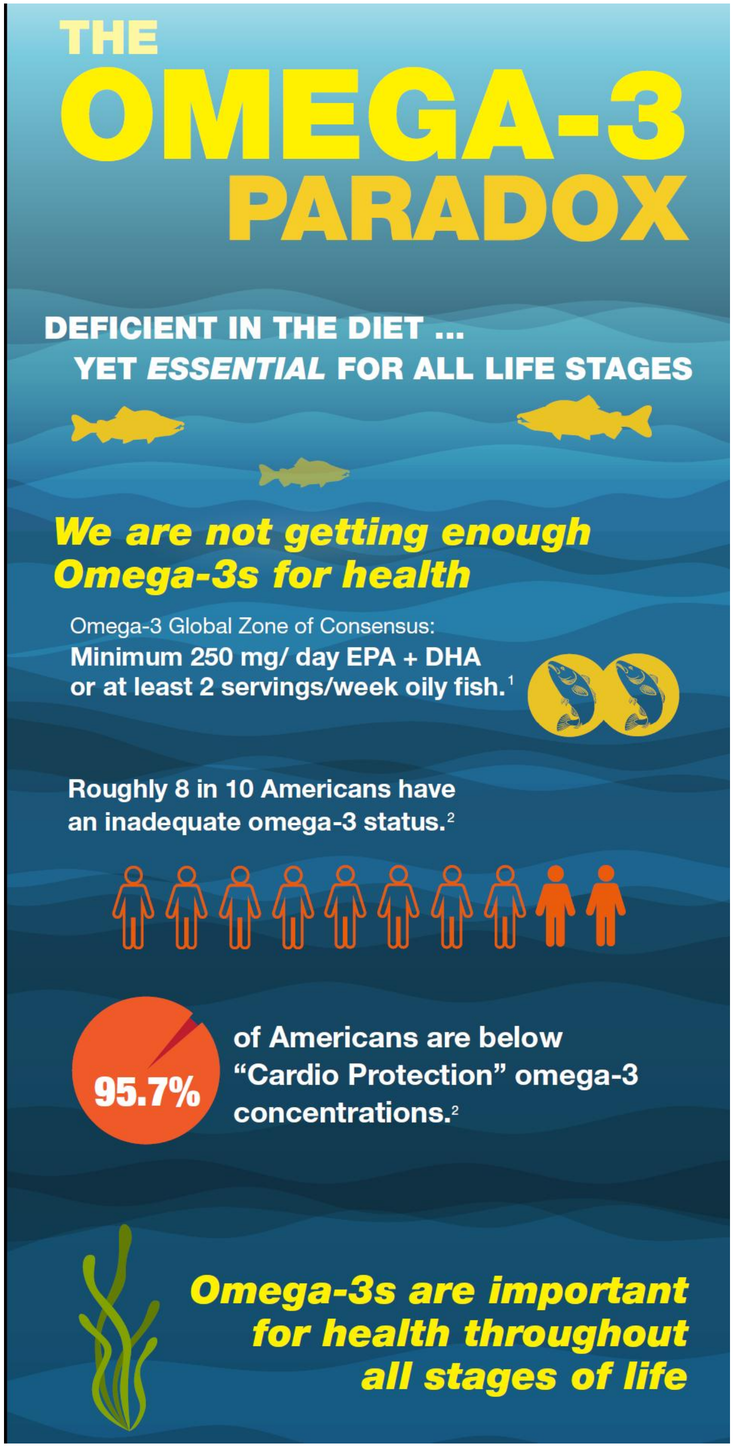

New!

Have your omega-3 status measured as part of our onsite wellness screening!

If you have questions, contact Michael McBurney, PhD
Telephone: 973-257-8232
Cellular: 862-222-3646
E-mail: Michael.McBurney@dsm.com

DSM

[1] Mozaffarian D and Wu J. 2011 J Am Coll Card 58:2047
[2] Murphy R et al. 2015. Nutrients 7:10282
