# Supplementary material for: Impact of Biological Feedback and Incentives on Blood Fatty Acid Concentrations, Including Omega-3 Index, in an Employer-Based Wellness Program
Source: Nutrients. 2017 Aug 5;9(8):842. doi: 10.3390/nu9080842 (PMC5579635; doi:10.3390/nu9080842)
Supplement: Supplementary file 1 [file nutrients-09-00842-s001.zip › Figure S2 Consent form.pdf]

INFORMED CONSENT TO TAKE PART IN A RESEARCH STUDY

TITLE OF STUDY: **Cross-sectional Survey of Omega-3 Fatty Acid Blood Levels**

INVESTIGATOR: **Michael McBurney, PhD, FACN**
CONTACT: **973-257-8232 (office), 862-222-3646 (mobile)**
Email: Michael.McBurney@dsm.com

SPONSOR: **DSM**

INTRODUCTION

You are being asked to participate in a research study. This form describes the purpose, procedures, benefits, risks, discomforts, and precautions of participating in this study. It also describes your right to not participate at any time.

DSM sells omega-3s and fish oils to the dietary supplement and food industries. Omega-3s are important for heart, brain, and joint health. Most Americans have low levels of omega-3s in their blood. Blood levels of omega-3s can be improved by simple dietary changes. The only way to know blood level of omega-3s is by measurement. The Omega-3 index, a measure of levels of omega-3s in blood, is determined in a single drop of blood obtained from a finger prick. According to the OmegaQuant LLC website, the Omega-3 Index test has been safely measured in more than 1,000,000 people.

Employees working at DSM workplaces with an onsite Healthyroads Wellness® biometrics screening program are being offered an opportunity to have their blood omega-3 levels measured at no charge.

DSM will not have access to any information which can directly link an employee's blood levels of omega-3s or survey responses with an employee; nor will any health data collected by Healthyroads Wellness® be communicated to DSM.

PURPOSE

- Determine the distribution of whole blood fatty acids, including the omega-3 index and related parameters, of the participants.
- Provide DSM employees information on their omega-3 levels in blood
- Descriptive statistics of the fatty acid profiles of participants by:
  - Self-reported omega-3 consumption
  - Sex
  - Age

BACKGROUND

- In the USA, DSM offers a comprehensive wellness program, Healthyroads Wellness®. Employees can earn incentives by completing certain actions, including biometric screening.

- DSM would like to offer participants of onsite Healthyroads Wellness® biometric screenings the opportunity to volunteer in an omega-3 research study to learn about the omega-3 fatty acid levels in their blood.

GENERAL INFORMATION ABOUT THE STUDY

- As part of the Healthyroads Wellness® biometric screening, a finger stick is required to measure blood cholesterol levels. In the blood cholesterol measurement, some blood is discarded. This 'extra' blood can be used to test the omega-3 fatty acid level.
- For participants agreeing to participate in the omega-3 research study, a blood drop will be applied to a filter paper. Their completed Omega-3 Test Request form, signed Informed Consent form and Filter Paper with whole blood sample will be stapled together and sent to an outside laboratory, OmegaQuant LLC, for blood fatty acid and data analysis.
- Each participant will receive an Omega-3 Index Test report by email directly from OmegaQuant LLC within a week of receipt of the blood sample. The Omega-3 Index Test results are accompanied by information on the health benefits of omega-3s and dietary sources to increase blood levels.
- After removing personal information, OmegaQuant LLC will share blood fatty acid and dietary data with DSM for statistical and reporting purposes.
- DSM will be unable to link data with any employee.

STUDY PROCEDURES

General overview (as applicable):

- A Healthyroads Wellness® biometrics screening includes a finger stick for blood cholesterol measurement. When measuring blood cholesterol, some blood is discarded. For those volunteering to participate, blood obtained from the finger stick for cholesterol measurement will be used to measure blood levels of omega-3 fatty acids.

Details of Procedures:

- Email messages and posters announcing onsite HealthyRoads Wellness biometric screening clinics will inform ~1,800 DSM employees at 19 locations of the opportunity to participate in an omega-3 research study and have their blood omega-3 levels measured.
- Participants attending a Healthyroads Wellness® biometrics screening clinic will be invited to participate. If they agree and sign an Informed Consent form, they need to complete an Omega-3 Index Test Request form, and write their name, date of birth and collection date on an OmegaQuant LLC filter paper. The request form includes some dietary questions about fish/meat consumption.
- Omega-3 research participants will proceed to the biometric screening station where blood obtained from the finger stick for blood cholesterol measurements will be applied to a filter paper by a Healthyroads Wellness® professional.
- The filter paper, the Omega-3 Index Test Request, and Informed Consent form will be stapled together, packaged with all other samples obtained at that location, and shipped by DSM to OmegaQuant LLC.
- Within about a week of receiving the blood sample, OmegaQuant LLC will email the Omega-3 Test results directly to each participant.
- There are no other obligations.

WHAT IS EXPERIMENTAL

The experimental purpose is to:

- Provide DSM with insights on omega-3 fatty acid levels by self-reported omega-3 consumption, sex, and age.
- Provide DSM employees with information on their omega-3 status.

RISKS

- Minimal risk of discomfort from a finger stick for cholesterol measurement by a trained biometrics screener.

NEW FINDINGS

- Cross-sectional analysis of whole blood fatty acids and self-reported omega-3 consumption, sex and age will be published and made publicly available.

BENEFITS

- Participating in this study may or may not provide a direct benefit to you.

CONTACT INFORMATION IN THE EVENT OF AN EMERGENCY

- For immediate reactions to the finger stick, follow-up with onsite biometrics screener.

RESEARCH RELATED INJURY (required only if study is more than minimal risk)

- None anticipated from a finger stick.

ALTERNATIVE TREATMENT

- Not applicable

COSTS OF PARTICIPATION

- None to the participants

COMPENSATION FOR PARTICIPATION

- No compensation for participants
- A limited number of coupons for a discount (value <$7) on a 90 day supply of omega-3 capsules will be available at some of the onsite screenings. These coupons will be offered to participants in order to determine rates of coupon redemption. If a coupon is offered at a biometrics screening site, a single coupon will be offered to each participant at that location. The coupon will be offered after the finger stick procedure so that it is not an incentive for participation.

VOLUNTARY PARTICIPATION

- Your participation in this study is voluntary. Your refusal to participate in the study will involve no penalty or loss of benefits to which you are entitled.

REMOVAL FROM THE STUDY

Your study doctor may end your participation in this study for any of the following reasons:

- If the study is cancelled by the sponsor, the New England Independent Review Board or by the FDA;
- For administrative reasons.

CONFIDENTIALITY

- The signed Informed Consent form, Omega-3 Test Request form, and Filter Paper with dried blood spot will be stapled together and shipped in bulk at the end of the day to OmegaQuant LLC which will retain these records.
- Participants will receive their Omega-3 Test report by direct email from OmegaQuant LLC.
- OmegaQuant LLC will collate responses from Omega-3 Test Request form and blood fatty acid analysis into an electronic database and assign a unique identification code for each individual.
- OmegaQuant LLC will remove personal information (name, birthdate, and email address) before sharing data (blood fatty acid levels and dietary survey responses) with DSM for statistical and reporting purposes.
- DSM will not have access to any information which can directly link an employee's blood levels of omega-3s or survey responses with an employee; nor will any health data collected by Healthyroads Wellness® be communicated to DSM.

Who will have access to your study and/or medical information?

Records of your participation in this study will be held confidential by OmegaQuant LLC so far as permitted by law. However, under certain circumstances, the Food and Drug Administration (FDA) and New England Independent Review Board (IRB) will be able to inspect and have access to confidential data that identifies you by name. Any publication or presentation of the data will not identify you. By signing this consent form, you authorize the release of your medical records to the FDA and the IRB.

QUESTIONS ABOUT THE STUDY

If you have questions, complaints, or concerns about the study, you can contact Michael I McBurney, PhD at (973)257-8232 (office) or at (862)222-3646 (mobile) or by email: Michael.McBurney@dsm.com.

If you have questions about your rights as a research subject, or other concerns about the research, you can contact the New England Independent Review Board (NEIRB) at 1-800-232-9570.

For Employees of DSM:

For Employees of «company»:

Your participation in this study is voluntary. You are free to withdraw your consent and discontinue participation in this study at any time without prejudice or penalty. Your decision to participate or not participate in this study will in no way affect your continued employment or your relationship with individuals who may have an interest in this study. _______initials

VOLUNTEER'S STATEMENT:

I agree that I have been given a chance to ask questions about this omega-3 research study where I self-report dietary practices and learn about the levels of omega-3 fatty acid levels in my blood. These questions have been answered to my satisfaction. I may contact ***Michael I McBurney, PhD, Office Tel: 973-257-8232, Mobile 862-222-3646, Email Michael.McBurney@dsm.com,*** if I have any more questions about this study. He is employed by ***DSM*** which is paying the costs to measure my omega-3 levels in blood.

My participation in this research project is voluntary. I may quit the study at any time without harming my future medical care or losing any benefits to which I might be entitled. If I have questions about my rights as a research subject, other concerns about the research, or I am unable to reach the investigator, I can contact:

New England Institutional Review Board

Telephone: 1-800-232-9570

By signing this form, I have not waived any of my legal rights.

I agree to participate in this study. I will be given a copy of this signed and dated form for my own records.

________________________________ __________

Study Participant (signature) Date

____

Print Participant’s Name

________________________________ __________

Witness (signature) Date

Approved by NEIRB on 4/5/2016
NEIRB Version 1.0
