# Supplementary material for: Impact of Biological Feedback and Incentives on Blood Fatty Acid Concentrations, Including Omega-3 Index, in an Employer-Based Wellness Program
Source: Nutrients. 2017 Aug 5;9(8):842. doi: 10.3390/nu9080842 (PMC5579635; doi:10.3390/nu9080842)
Supplement: Supplementary file 1 [file nutrients-09-00842-s001.zip › Figure S3 Test request form.pdf]

Space for Unique Identification Code

Omega-3 Index Test Request Form

1. Name ____

*** Must match the name on the sample collection booklet **

2. Results Email ______________________________________

3. Date of birth ______ ___ ______ Collection date ______ ___ ______
Month Day Year Month Day Year

4. Male __________ Female ____________

Estimate your consumption over the past 2 months of the following foods.
A serving is about the size of a pack of playing cards.

Servings

5. How many 3 oz. servings of the following fish do you eat monthly? ________

bluefish	herring	sardines
blue fin tuna	mackerel	salmon
cisco, smoked	pollock	whitefish

6. How many 3 oz. servings of the following fish do you eat monthly? ________

bass	mussels	squid
calamari	perch	swordfish
catfish	redfish	trout
drumfish	rockfish	tuna, canned (6oz can)
flounder	shark	whiting
grouper	snapper	
halibut	sole	

7. How many 3 oz. servings of the following fish/shellfish do you eat monthly? ________

carp	fish sticks	pompano
clams	haddock	scallops
cod	lobster	shrimp (14 med.)
crab	mullet	sturgeon
crayfish	oysters	
fish patties/squares	pike	

8. How many 3 oz. servings of liver (chicken, turkey or beef) do you eat monthly? ________

9. How many egg yolks do you eat weekly (including egg yolks used in cooking)? ________

10. How many 3 oz. servings of chicken, turkey or other poultry (not including livers) do you eat weekly? ________

11. Any omega-3 dietary supplements or functional foods (i.e. flax, fish oil, Neuromins, DHA Gold, high DHA eggs)?

amount or strength ________ frequency ________
milligrams per day
