# Supplementary figures and images for: Impact of Biological Feedback and Incentives on Blood Fatty Acid Concentrations, Including Omega-3 Index, in an Employer-Based Wellness Program
Source: Nutrients. 2017 Aug 5;9(8):842. doi: 10.3390/nu9080842 (PMC5579635; doi:10.3390/nu9080842)

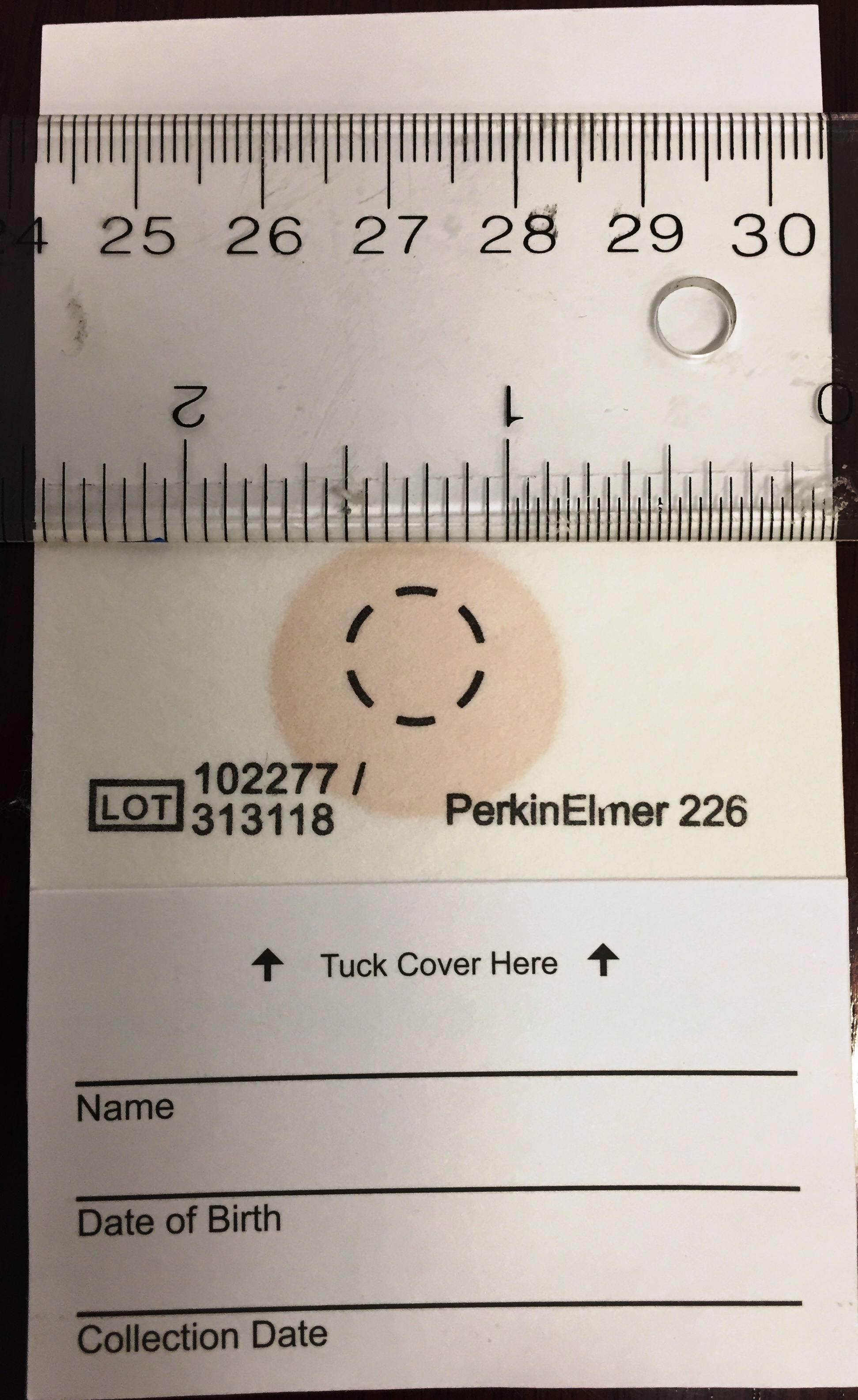

Supplement: Supplementary file 1 [file nutrients-09-00842-s001.zip › Figure S4 Filter paper for blood spot.jpg]
